# Inhibitory effects of ChondroT and its constituent herbs on RANKL-induced osteoclastogenesis

**DOI:** 10.1186/s12906-019-2737-8

**Published:** 2019-11-20

**Authors:** Rui Hong Guo, Seon-Jong Kim, Chan-hun Choi, Chang-su Na, Bok Yun Kang, Young Ran Kim

**Affiliations:** 1 College of Pharmacy and Research Institute of Drug Development, Chonnam National University, Gwangju, 500-757 Republic of Korea; 2 College of Korean Medicine, Dongshin University, 185 Geonjae-ro, Naju-si, Jeollanam-do 58245 Republic of Korea

**Keywords:** ChondroT, Osteoclast, Bone resorption, NF-κB, MAPKs

## Abstract

**Background:**

ChondroT is a complex herbal medicine consisting of water extracts of *Ostericum koreanum* (Maxim.) Kitag., *Lonicera japonica* Thunb., *Angelica gigas* Nakai, *Clematis manshurica* Rupr., and *Phellodendron amurense* Rupr. (6:4:4:4:3). Previous studies have reported that ChondroT possesses chondroprotective and anti-inflammatory, anti-osteoarthritic, and anti-hyperuricemic activities. The study is aim to demonstrate the effects of ChondroT and its five constituent herbs on receptor activator of NF-κB ligand (RANKL)-induced osteoclastogenesis and the underlying mechanisms.

**Methods:**

Osteoclastogenesis was identified in bone marrow-derived macrophages (BMDMs) by tartrate-resistant acid phosphatase (TRAP) staining assay, actin ring formation assay and the bone resorption assay. For the molecular mechanisms, activation of RANKL-induced NF-κB and MAPK signaling pathways and the expression levels of osteoclast-specific proteins were investigated by Western blotting. Cell viability was assessed by MTT assay. Actin ring formation and NF-κB translocation were evaluated by immunostaining.

**Results:**

ChondroT and each of its constituent herbs significantly suppressed osteoclast differentiation dose dependently, and decreased actin ring formation as well as bone-resorbing capacity. Mechanistically, ChondroT and its constituent herbs downregulated the expressional levels of osteoclast-specific proteins such as NFATc1, c-Fos, Cathepsin K, and matrix metalloproteinase 9 (MMP9) by suppressing NF-κB translocation to nucleus and MAPKs phosphorylation at different levels. Compared to its five constituent herbs, ChondroT exhibited the best inhibitory efficiency against osteoclastogenesis.

**Conclusions:**

Taken together, ChondroT has anti-osteoclastogenesis properties by inhibiting NF-κB and MAPKs pathways. It could be considered as a potential therapeutic candidate for the treatment of osteoclast-related bone diseases.

## Background

Bone homeostasis is an important physiological process that involved in the functional balance between bone depositing osteoblasts and bone resorption osteoclasts. Differentiation of these two types of cells are crucial to maintain the normal physiology of the bones [1]. Osteoclasts are bone resorption multinucleated cells (MNCs), derived from hematopoietic stem cells of monocyte/macrophage lineage [2]. The receptor activator of nuclear factor κ-B (NF-κB) ligand (RANKL) and the macrophage colony-stimulating factor (M-CSF) have been reported to be crucial for regulating osteoclast differentiation. RANKL is important for the differentiation and activation of osteoclasts, and M-CSF is responsible for survival and proliferation of osteoclasts precursors [3, 4]. Binding of RANKL to RANK receptor could lead to the recruitment of tumor necrosis factor (TNF) receptor-associated factor 6 (TRAF6), and consequently activates not only NF-κB, but also mitogen-activated protein kinases (MAPKs) [5–7]. This signaling cascade activates the nuclear factor of activated T cells c1 (NFATc1) and a member of activator protein 1 (AP-1) family c-Fos [8, 9]. Subsequently, RANKL upregulates osteoclast-specific genes such as matrix metalloprotease-9 (MMP9) and Cathepsin K [10]. These signal molecules ultimately lead to the survival, activation, and differentiation of actin rings and bone resorption by osteoclasts [11]. Abnormal differentiation and dysfunction of osteoclasts, especially the excessive activity of osteoclasts, can lead to osteoporosis, osteoarthritis, and rheumatoid arthritis [12].

In recent years, osteoporosis has been a serious public health problem. However, some clinically available therapies are effective but are limited due to their side effects [13]. Natural products are studied as important sources of therapeutic drug molecules. We have previously reported that Ganghwaljetongyeum (GHJTY), a traditional decoction composed of 18 herbs, could be used for the treatment of fever, swelling, limitation of motion, joint pain, and inflammatory processes related to arthritis [14, 15]. We selected five effective herbal constituents from GHJTY with greatest potential to enhance the efficacy and convenience of drug prescription through bioinformatics analysis and pharmacologic activity tests [16]. The resulting concoction named as ChondroT, which comprised water extracts of *Ostericum koreanum* (Maxim.) Kitag. (O), *Lonicera japonica* Thunb. (L), *Angelica gigas* Nakai (A), *Clematis manshurica* Rupr. (C), and *Phellodendron amurense* Rupr. (P) in a 6: 4: 4: 4: 3 ratio [17]. ChondroT exhibited more significant chondroprotective effects and anti-inflammatory processes related to arthritis than GHJTY did [17]. ChondroT also significantly demonstrated the efficacy of anti-osteoarthritis in a rat model of osteoarthritis induced by monosodium iodoacetate- or collagenase [14, 18]. In addition, the efficacy and safety of ChondroT on knee-osteoarthritis were evaluated by randomized, double-blind, placebo-controlled, multicenter clinical trials [19]. Recently, we demonstrated that ChondroT exhibited the anti-hyperuricemic effects by regulating xanthine oxidase activity and kidney mouse urate transporter 1 in a potassium oxonate-induced hyperuricemic mouse model [20]. To further investigate the efficacy and mechanism of ChondroT as a therapeutic potential herbal medicine, we evaluated its function on bone disease in this study. Among its five constituent herbs, *Ostericum koreanum* (Maxim.) Kitag., *Angelica gigas* Nakai and its major active decursin are found to possess anti-osteoclastogenic activity in bone marrow cells isolated from mice [21–23]. Recently, the component phellodendrine from *Cortex Phellodendri Chinensis* has been reported to have an obvious inhibitory effect on osteoclast differentiation and function [24]. Therefore, the complex herbal medicine ChondroT has potential beneficial effects against osteoclastogenesis. The present study is aim to investigate anti-osteoclastogenic effects of ChondroT and its five constituent herbs in RANKL-activated primary precursor cells and the underlying signaling pathways involved.

## Methods

### Plant materials

ChondroT was prepared using a previously described method [17]. We purchased five herbal medicines from Omniherb (Yeongcheon, Korea) as shown in Table 1. Professor Jong-Kil Jeong from the Department of Herbology, college of Oriental Medicine of Dongshin University confirmed their origins taxonomically. Voucher specimens (KYR2014–020) were deposited at the college of Pharmacy, Chonnam National University.

**Table 1:** Constituents of ChondroT

Latin name	Scientific name	ratio	source
Osterici Radix	*Ostericum koreanum* (Maxim.) Kitag.	6	Korea
Lonicerae Folium	*Lonicera japonica* Thunb.	4	China
Angelicae Gigantis Radix	*Angelica gigas* Nakai	4	Korea
Clematidis Radix	*Clematis manshurica* Rupr.	4	China
Phellodendri Cortex	*Phellodendron amurense* Rupr.	3	China

Stock solutions (100 mg/mL) of all the herbs were diluted using phosphate buffered saline (PBS), sterilized by filtration, and diluted to the working concentration with PBS.

### Cell culture and reagents

Recombinant mRANKL was acquired from R&D systems (Minneapolis, MN, USA). Alpha-minimum essential medium (α-MEM) was obtained from Welgene (South Korean). Fetal bovine serum (FBS), trypsin-EDTA, and penicillin/streptomycin were purchased from Gibco (Grand island, NY, USA). Antibodies specific to c-Fos (sc-271243), NFATc1 (sc-17834), Cathepsin K (sc-48353), MMP9 (sc-393859), and GAPDH (sc-25778) were obtained from Santa Cruz Biotechnology. In addition, NF-κB p65 (sc-372), LaminB (sc-6216), β-actin (sc-47778), JNK (sc-571) were also purchased by Santa Cruz Biotechnology. ERK (#4695), p-ERK (#9101), p38 (#9212), p-p38 (#9211), p-JNK (#9251) were obtained by Cell signaling Tech. The murine fibroblastic cell line L929 was used as a source of M-CSF. L929 cells were purchased from ATCC, and cultured in Iscove’s Modified Dulbecco’s Medium (IMDM, Gibco, Grand island, NY, USA) containing 10% FBS and 1% penicillin/streptomycin until confluent. L929 cell free conditioned medium was harvested, filtered with 0.45 μm filter, and stored at − 80 °C until use. Bone marrow-derived macrophages (BMDMs) were cultured in α-MEM with 10% of FBS and 1% of penicillin/streptomycin containing M-CSF, which was secreted by L929 cells and was used in the form of L929-conditioned medium.

### Animals

Animal experiments were approved by the Institutional Animal Care and Use Committee (IACUC) of Chonnam National University (Approal number: CNU IACUC-YB-2018-71). Five-week-old C57BL/6 male mice were bought from Damool Science (Daejeon, Korea) and housed in a specific pathogen free facility with a controlled environment at a temperature of 22–24 °C and a humidity of 55–60% with 12 h day/night cycles.

### Osteoclast differentiation

Mice were anesthetized with Carbon dioxide (CO_2_), and then were sacrificed by cervical dislocation in accordance with IACUC guidelines. Primary bone marrow (BM) was isolated from the femurs and tibiae of 5-week-old C57BL/6 J mice, followed by culture in a 150 mm dish with α-MEM medium supplemented with 10% FBS and 1% penicillin/streptomycin containing 30% of L929-conditioned medium for 4 days to induce differentiation into BMDMs. Adherent cells (2 × 10^4^ cells/well) were plated into 96-well plates, and cultured for 4 days in the presences of L929 secreted M-CSF and 100 ng/mL of RANKL with or without ChondroT or its constituent herbs. After 4 days, remove the culture medium and washed each well with 100 μL of PBS. Cells were then fixed with 10% of paraformaldehyde for 10 min, permeabilized with 0.1% of Triton X-100 for 1 min, and stained with TRAP staining kit (Kamiya Biomedical Company, Seattle, WA, USA) following the manufacturer’s instructions. TRAP-positive cells with more than three nuclei were considered as osteoclasts. 30 μL of culture supernatant was dispensed into a 96-well plate and 170 μL of chromogenic substrate/tartrate-containing buffer was added at 37 °C for 3 h. TRAP activity in osteoclast culture supernatant was determined at 540 nm.

### Measurement of cell viability

BMDMs (4 × 10^4^ cells/well) were plated in a 96-well plate (SPL life Sciences Co., Pocheon, Korea) and cultured overnight in the presence of 30% of L929-conditioned medium in a humidified 37 °C incubator. Cells were treated with ChondroT or its constituent herbs for 72 h. After 72 h, cells were treated with 3-(4,5-dimethylthiazol-2-yl)-2,5-diphenyltetrazolium bromide (MTT) (5 mg/10 mL) solution for 4 h. Remove the MTT solution and dissolve the purple colored formazan crystals using 200 μL of dimethyl sulphoxide (DMSO). Absorbance was read at 570 nm by a microplate reader (BioTek, Winooski, VT, USA).

### Actin ring staining

BMDMs (3 × 10^4^ cells/well) were seeded into 8-well glass chamber plates (Thermo Fisher Scientific, USA) in the presence of 30% of L929-conditioned medium and 100 ng/mL of RANKL with or without ChondroT or its constituent herbs for 4 days. Cells were then fixed with 4% of paraformaldehyde (Molecular Probes, OR, USA) and permeabilized using 0.1% of triton. After blocking, actin was visualized by incubating cells with Alexa Fluor 488-conjugated phalloidin. Cells were mounted with anti-fade reagent with DAPI (Molecular Probes). Fluorescence images were photographed by a fluorescence microscope (Nikon DS-Ri2 microscope camera, Tokyo, Japan).

### Bone resorption assay

BMDMs (2.5 × 10^5^ cells/well) were plated into 24-well Osteo assay surface plates (Corning Incorporated, ME, USA) with α-MEM containing 30% of L929-conditioned medium, and then incubated with 100 ng/mL of RANKL with or without ChondroT or its constituent herbs for 4 days. Cells were lysed with 20% SDS at room temperature for 15 min, washed with distilled water for 3 times, and air dried for 3 ~ 5 h. Resorption pits on the Osteo assay plates were photographed. Resorbed areas were analyzed with ImageJ program [25].

### NF-κB staining

BMDMs (3 × 10^4^ cells/well) seeded into 8-well glass chamber plates were incubated overnight in the presence of 30% of L929-conditioned medium. Cells were pretreated with ChondroT or its constituent herbs for 4 h, and were stimulated with 100 ng/mL of RANKL for 30 min. Cells were then fixed with 4% of paraformaldehyde, and permeabilized using 0.1% of triton. A polyclonal anti-NF-κB p65 antibody (Invitrogen, Carlsbad, MA, USA) and an Alexa Fluor 488-conjugated anti-rabbit IgG second antibody (Molecular Probes Invitrogen, MA, USA) were used for the detection of NF-κB p65 protein. Cells were then mounted with anti-fade reagent with DAPI. Bay 11–7082 was used as a positive control of NF-κB inhibitors. Fluorescence images were photographed with a fluorescence microscope (Nikon DS-Ri2 microscope camera, Tokyo, Japan) [26].

### Western blot analysis

BMDMs were incubated with 30% of L929-conditioned medium and 100 ng/mL of RANKL with or without ChondroT or its constituent herbs for indicated time. Cell lysates were quantified with Bradford’s reagent (Bio-Rad, USA). Equal concentrations of proteins were subjected to 10% SDS-PAGE, and were transferred onto PVDF membranes (Millipore Ltd., Carrigtwohill, Germany). After blocking, membranes were incubated with antibodies specific to osteoclast related proteins (c-Fos, NFATc1, MMP9, Cathepsin K, GAPDH, 1:1000, Santa Cruz, CA, USA), MAPKs signals [JNK (1:500, Santa Cruz, CA, USA) and (p-JNK, ERK, pERK, p38, p-p38, 1:500, Cell signaling Tech, MA, USA)], and NF-κB signals (NF-κB p65, LaminB, β-actin, 1:1000, Santa Cruz, CA, USA), and then incubated with HRP-conjugated secondary antibodies. Immunoreactive proteins were detected with an ECL Western blot detection system (Advansta, Menlo Park, CA, USA).

### Statistical analysis

All results are presented as means ± SEM. Statistical comparisons were evaluated using ANOVA. *P* value < 0.05 was considered statistically significant. All the data were acquired in quadruplicate and the experiments were repeated at least three times. Results are shown from the representative experiments.

## Results

### Effects of ChondroT and its constituent herbs on RANKL-induced osteoclast differentiation

**Fig. 1:**
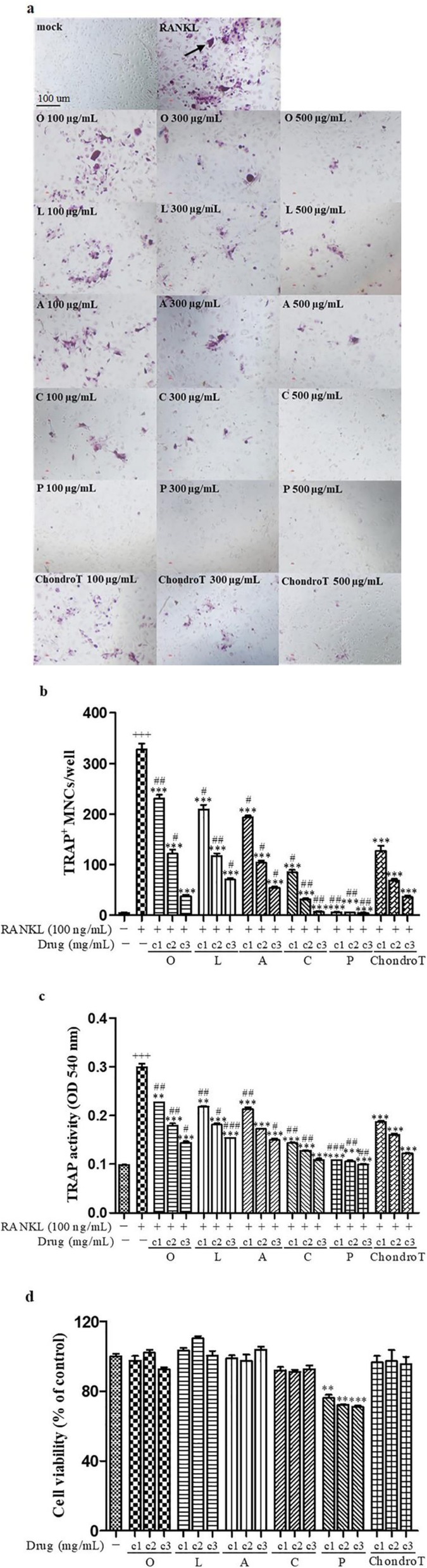
Effects of ChondroT and its constituent herbs on RANKL-induced osteoclast differentiation in BMDMs. **a** TRAP-positive stained cells were stained with TRAP staining kit. **b** TRAP-positive stained MNCs with more than three nuclei were counted as osteoclasts. **c** TRAP activity in osteoclast culture supernatant was determined at 540 nm. ^**^P < 0.01; ^***^P < 0.001 versus RANKL group; ^+++^*P* < 0.001 versus control group; ^#^*P* < 0.05; ^##^*P* < 0.01; ^###^*P* < 0.001 versus ChondroT group. **d** BMDMs pretreated with ChondroT or its constituent herbs for 72 h were treated with MTT solution for 4 h, and then were dissolved in DMSO. Absorbance was read at 570 nm. ^**^P < 0.01; ^***^P < 0.001 versus control group. Abbreviations: c1: 0.1 mg/ mL; c2: 0.3 mg/ mL; c3: 0.5 mg/mL

To investigate effects of ChondroT and its five constituent herbs on osteoclastogenesis, we examined osteoclast formation using bone marrow cells. Osteoclasts were characterized by monitoring the number of MNCs that showing positive staining of TRAP, an important enzyme marker of osteoclasts. RANKL stimulated the differentiation of BMDMs into TRAP-positive stained MNCs. However, ChondroT and its five constituent herbs significantly inhibited the TRAP activity and MNCs formation (Fig. 1 a-c). To investigate whether such suppression of osteoclastogenesis by ChondroT and its constituent herbs was due to potential toxicity of these products, we conducted cell viability assay. Results showed ChondroT and its constituent herbs had no cytotoxic effect at tested concentration except *Phellodendron amurense* Rupr. (Fig. 1 d). To further identify the effect of *Phellodendron amurense* Rupr. on osteoclastogenesis without cytotoxicity, we conducted TRAP staining and MTT assay at the lower concentration. Results showed that *Phellodendron amurense* Rupr. significantly inhibited the RANKL-induced TRAP activity without any cytotoxicity (Additional file 1). Notably, ChondroT and its two constituent herbs *Clematis manshurica* Rupr. and *Phellodendron amurense* Rupr. showed better inhibitory effects on RANKL-induced osteoclast activation.

### Effects of ChondroT and its constituent herbs on RANKL-induced actin rings and bone resorption in BMDMs

**Fig. 2:**
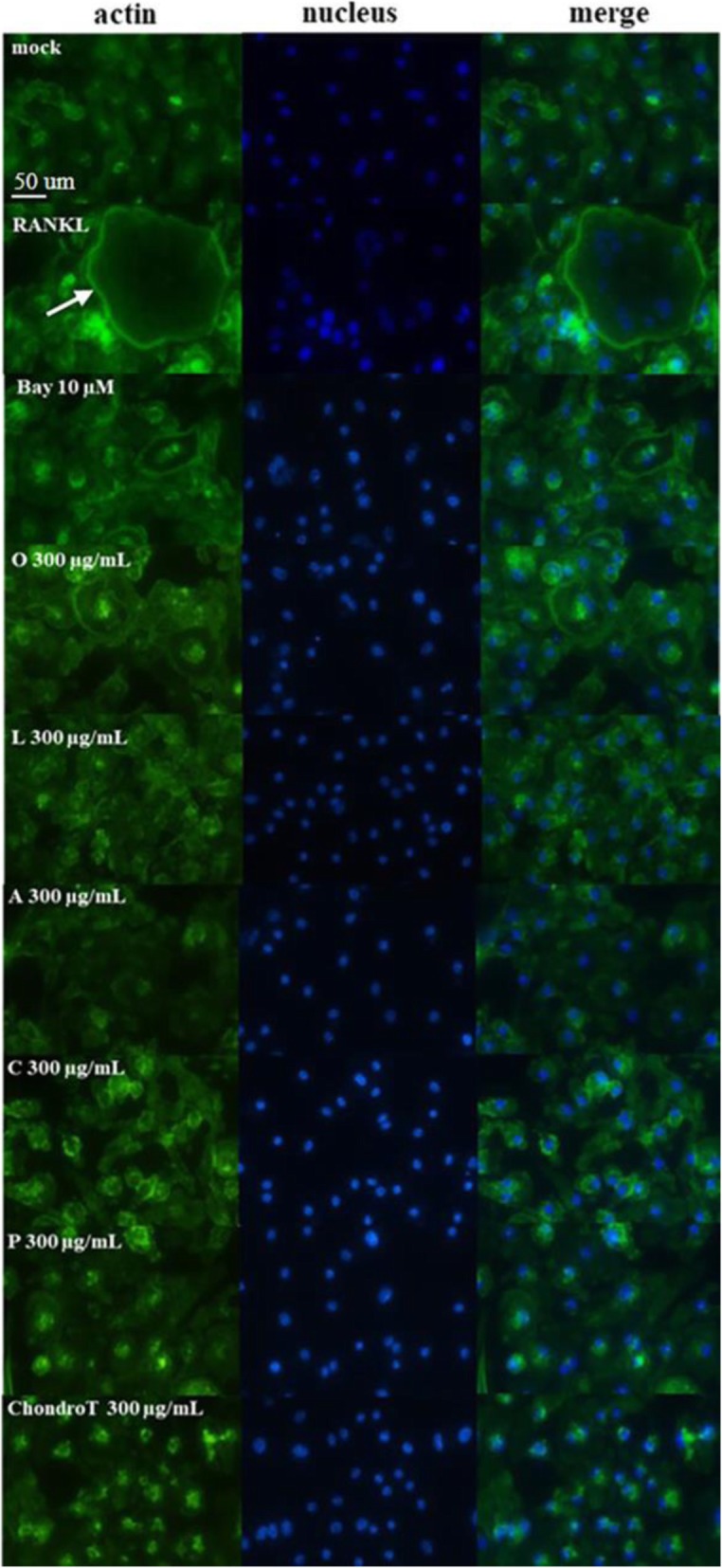
Effects of ChondroT and its constituent herbs on RANKL-induced actin rings in BMDMs. BMDMs were cultured in the presence of 30% of L929-conditioned medium and 100 ng/mL of RANKL with or without ChondroT or its constituent herbs for 4 days. Cells were stained with Alexa Fluor 488-conjugated phalloidin, and mounted with anti-fade reagent with DAPI. Bay 11–7082 was used as a positive control. Fluorescence images were photographed by a fluorescence microscope

**Fig. 3:**
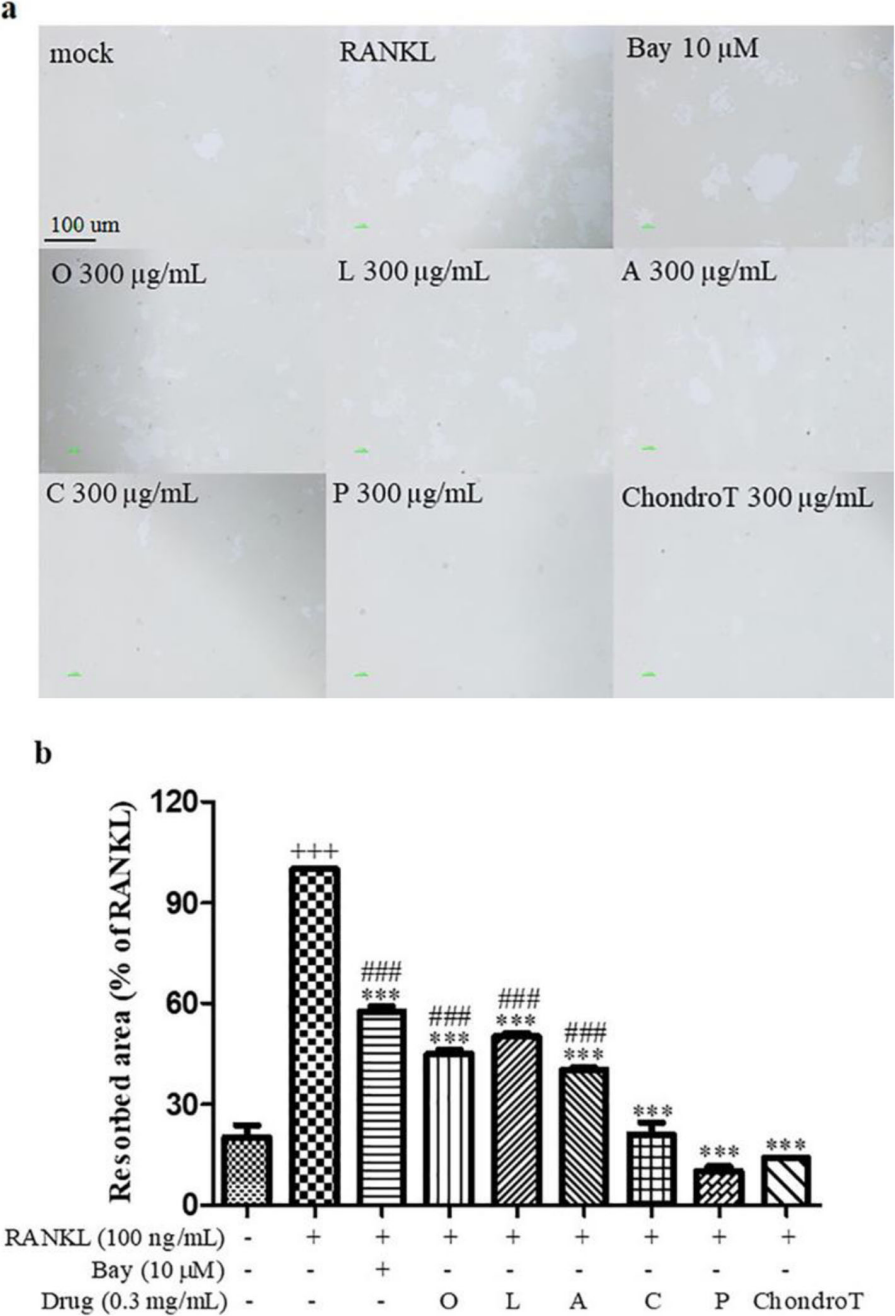
Effects of ChondroT and its constituent herbs on RANKL-induced bone resorption in BMDMs. BMDMs plated into Osteo assay surface plates were differentiated into osteoclasts. Cells were lysed with 20% SDS for 15 min and subsequently dried. **a** Resorption pits on the Osteo assay plates were photographed. **b** Resorbed areas were analyzed with ImageJ program. ^***^P < 0.001 versus RANKL group; ^+++^*P* < 0.001 versus control group; ^###^*P* < 0.001 versus ChondroT group

Actin ring formation is crucial for bone resorption by osteoclasts. Thus, we next determined effects of ChondroT and its five constituent herbs on the formation of actin ring structure and bone resorption ability. Control osteoclasts integrated actin ring structures and displayed a distribution of phalloidin-positive actin to the ventral side of cells. However, treatment with ChondroT or its five constituent herbs abolished actin ring structures in RANKL-induced cells (Fig. 2). Subsequently, we determined bone resorption activity using calcium phosphate-coated plates. We found that ChondroT and its five constituent herbs at a concentration of 300 μg/mL significantly decreased the number and size of bone resorption pits produced by mature osteoclasts especially ChondroT and its two constituent herbs *Clematis manshurica* Rupr. and *Phellodendron amurense* Rupr. (Fig. 3).

### Effects of ChondroT and its constituent herbs on RANKL-mediated osteoclast-specific protein expression in BMDMs

**Fig. 4:**
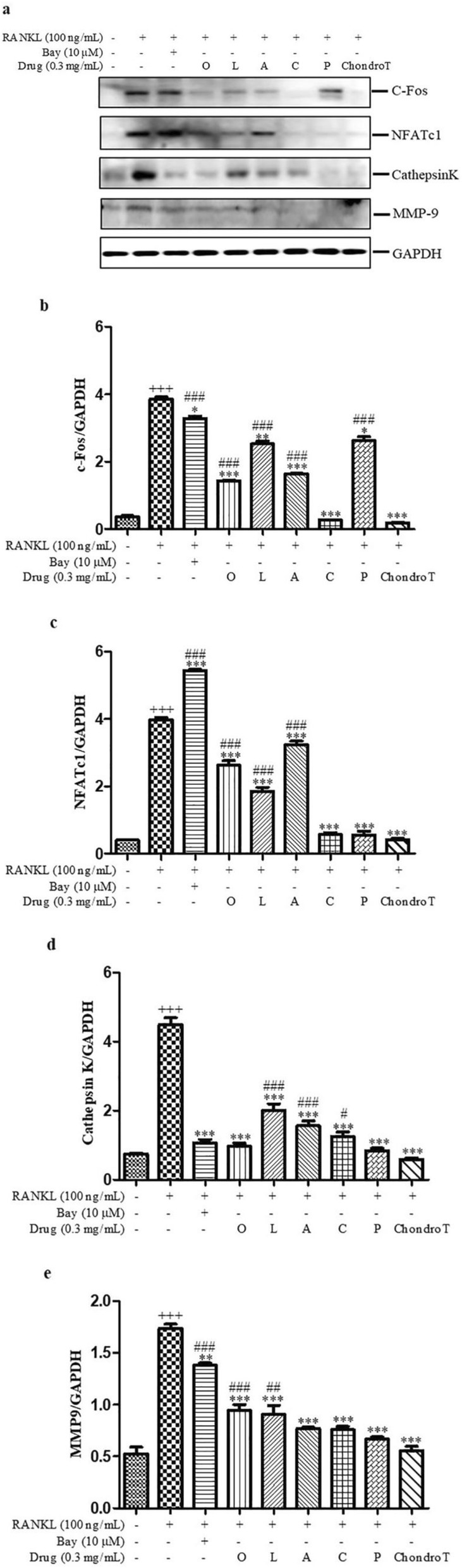
Effects of ChondroT and its constituent herbs on RANKL-mediated osteoclast-specific protein expression in BMDMs. BMDMs were pretreated with or without ChondroT or its constituent herbs for 4 h, and then stimulated with 100 ng/mL of RANKL for 48 h. **a** Expression levels of c-Fos, NFATc1, Cathepsin K, and MMP9 were determined by Western blotting. GAPDH was used as the loading control. Results are the representative of three independent experiments. **b-e** Protein levels from three independent experiments were quantified using ImageJ software. ^*^P < 0.05; ^**^P < 0.01; ^***^P < 0.001 versus RANKL group; ^+++^*P* < 0.001 versus control group; ^#^*P* < 0.05; ^##^*P* < 0.01; ^###^*P* < 0.001 versus ChondroT group

To further characterize the inhibitory effect of ChondroT and its five constituent herbs on RANKL-induced osteoclasts formation, we examined the expression levels of various osteoclast-specific proteins. RANKL exhibited the significant increase on the expression levels of these proteins during the process of osteoclast formation. ChondroT and its five constituent herbs downregulated expression levels of c-Fos and NFATc1 known to be associated with osteoclast differentiation. Moreover, MMP9 and Cathepsin K known to be associated with bone resorption activity were also significantly reduced. Among these herbs, ChondroT and its constituent herb *Clematis manshurica* Rupr. showed better inhibitory effects on various osteoclast-specific proteins (Fig. 4).

### Effects of ChondroT and its constituent herbs on RANKL-mediated NF-κB activation in BMDMs

**Fig. 5:**
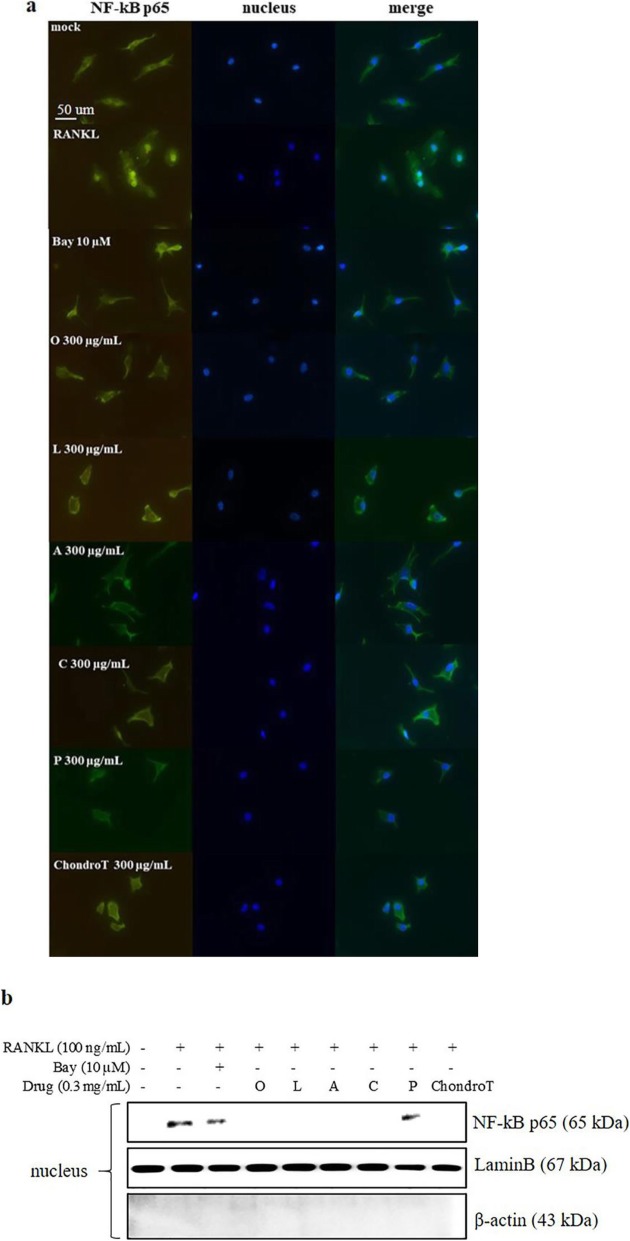
Effects of ChondroT and its constituent herbs on RANKL-mediated NF-κB activation in BMDMs. BMDMs were pretreated with ChondroT or its constituent herbs for 4 h prior to stimulation with 100 ng/mL of RANKL for 30 min. **a** NF-κB p65 protein was detected with a polyclonal-anti-NF-κB p65 antibody and Alexa Fluor 488-conjugated anti-rabbit IgG antibody, and then mounted with anti-fade reagent with DAPI. Bay 11–7082 was used as a positive control. **b** Nuclear fractions were isolated from BMDMs. Protein levels of NF-κB p65 in the nuclear fractions was detected by Western blotting

To explore the mechanisms by which ChondroT and its five constituent herbs inhibited RANKL-induced osteoclastogenesis, we first investigated the NF-κB signaling pathway, which has been reported to be crucial in RANKL-induced osteoclastogenesis. As shown in Fig. 5 a, RANKL caused NF-κB translocation from the cytosol to nucleus, which was suppressed by ChondroT and its five constituent herbs. Furthermore, we isolated the nuclei from BMDMs and determined NF-κB translocation by Western blotting. As shown in Fig. 5 b, RANKL-induced NF-κB translocation was remarkably decreased by treatment with ChondroT and its constituent herbs *Ostericum koreanum* (Maxim.) Kitag., *Lonicera japonica* Thunb., *Angelica gigas* Nakai, *Clematis manshurica* Rupr. although RANKL-induced NF-κB translocation was only slightly decreased by the treatment of *Phellodendron amurense* Rupr. It is currently clear that ChondroT and its five constituent herbs exhibiting anti-osteoclastogenic activity are involved in NF-κB signaling pathway.

### Effects of ChondroT and its constituent herbs on RANKL-induced MAPK activation in BMDMs

**Fig. 6:**
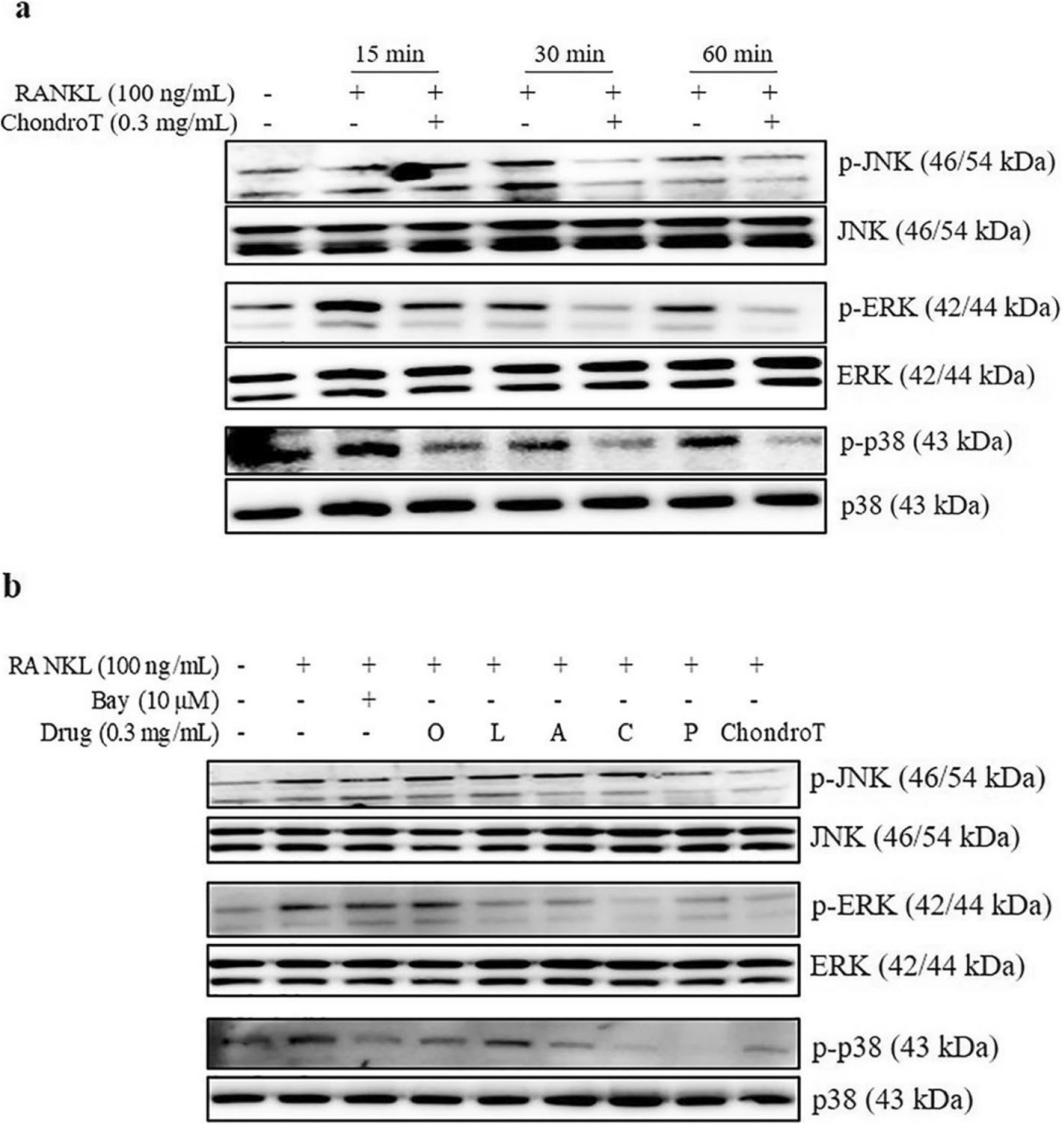
Effects of ChondroT and its constituent herbs on RANKL-induced MAPKs activation in BMDMs. **a** BMDMs were pretreated with ChondroT for 4 h prior to stimulation with 100 ng/mL of RANKL at the indicated time. **b** Cells were treated with ChondroT or its constituent herbs for 4 h prior to stimulation with 100 ng/mL of RANKL for 30 min. Whole cell lysates were used for Western blot analysis. Phosphorylated MAPKs were detected with antibodies against phospho-ERK, phospho-JNK or phospho-p38. Membranes were stripped and reassessed with antibodies targeting non-phosphorylated MAPKs

In addition to NF-κB signaling activation, MAPKs also plays an important role in osteoclastogenesis. To determine the appropriate RANKL stimulation time, we stimulated BMDMs with RANKL for indicated time, and found that ChondroT showed the best efficiency at 30 min (Fig. 6 a). As shown in Fig. 6 b, RANKL stimulation for 30 min resulted in higher phosphorylations of JNK, ERK, and p38. In contrast, treatment with five constituent herbs showed different degrees of inhibitions on RANKL-induced MAPK activation. Interestingly, ChondroT remarkably reduced RANKL-induced phosphorylations of all the three MAPK kinases.

## Discussion

Increased RANKL activity always leads to excessive osteoclast formation and bone resorption, which could result in a range of bone diseases such as osteoporosis, Paget’s disease, rheumatoid arthritis, and bone metastases [27]. Current clinically available therapies for bone diseases are effective but have some limitations and side-effects. Therefore, the development of novel alternative drugs is required [28]. Recently, there is a growing interest in natural product for the treatment of bone-related disorders. Previous studies have reported that *Ostericum koreanum* (Maxim.) Kitag. and *Angelica gigas* Nakai possess anti-osteoclastogenic properties [21, 22]. Hence, complex herbal medicine ChondroT might have beneficial effects on bone health. In this study, we found for the first time that ChondroT could inhibit RANKL-induced osteoclast activity in vitro without affecting the cell viability even at the highest dose of 500 μg/mL. This has essential therapeutic implications for treating bone diseases, since nonspecific cellular cytotoxicity might result in unwarranted side effects in patients.

Firstly, osteoclastogenesis was identified by TRAP staining, actin-ring formation and bone resorption in BMDMs, which showed ChondroT and its five constituent herbs could be used as potentially novel therapies for osteoclast-related bone disease (Fig. 1-3). Among these herbs, we found that ChondroT and its two constituent herbs *Clematis manshurica* Rupr. and *Phellodendron amurense* Rupr. showed better inhibitory effects on osteoclastogenesis. Subsequently, we researched mechanisms that inhibit osteoclast formation and function, particularly with regard to NF-κB pathway, MAPK pathway, and NFATc1. NF-κB, activated by RANKL, is an important osteoclastogenesis regulator. Binding of RANK with RANKL results in NF-κB release and its translocation from the cytosol to nucleus [29]. MAPKs (ERKs, JNKs, p38) are essential for RANK-activated osteoclast differentiation. ERK is reported to be important for the survival of osteoclasts. p38 and JNK are reported to be phosphorylated in response to RANKL activation [30]. Upon activation with RANKL, treatment with five constituent herbs showed different degrees of inhibitions on the activation of NF-κB and MAPKs. However, ChondroT remarkably suppressed the activations of both NF-κB and MAPKs signaling pathways (Fig. 5-6), which subsequently leads to the attenuation of NFATc1 and c-Fos. NFATc1 and c-Fos are two crucial transcription factors that regulate osteoclast differentiation [31]. Furthermore, RANKL upregulated osteoclast-specific genes Cathepsin K and MMP9, which were reduced by ChondroT and its five constituent herbs (Fig. 4). Cathepsin K and MMP9 are involved in the degradation of matrix proteins during bone resorption [32].

The studies discussed in this current work demonstrated the anti-osteoclastogenic effects of ChondroT and its constituent herbs in BMDMs. To further confirm the osteoprotective effect of ChondroT and its constituent herbs, the osteoprotective effect in vivo should be investigated. As far as we know, almost no relevant animal experiments of the five constituent herbs were conducted. Previous studies have reported that ultrafine *Angelica gigas* Nakai powder possesses the anti-osteoporosis properties in ovariectomized rats [33]. Decursin, the major active compound of *Angelica gigas* Nakai root, exhibited inhibitory effects on LPS-induced bone erosion in vivo [23]. In this study, ChondroT exhibited the significant inhibitory effect on osteoclastogenesis which indicates there exists an ample possibility that ChondroT may possess the ability of anti-osteoprotective in vivo.

## Conclusions

**Fig. 7:**
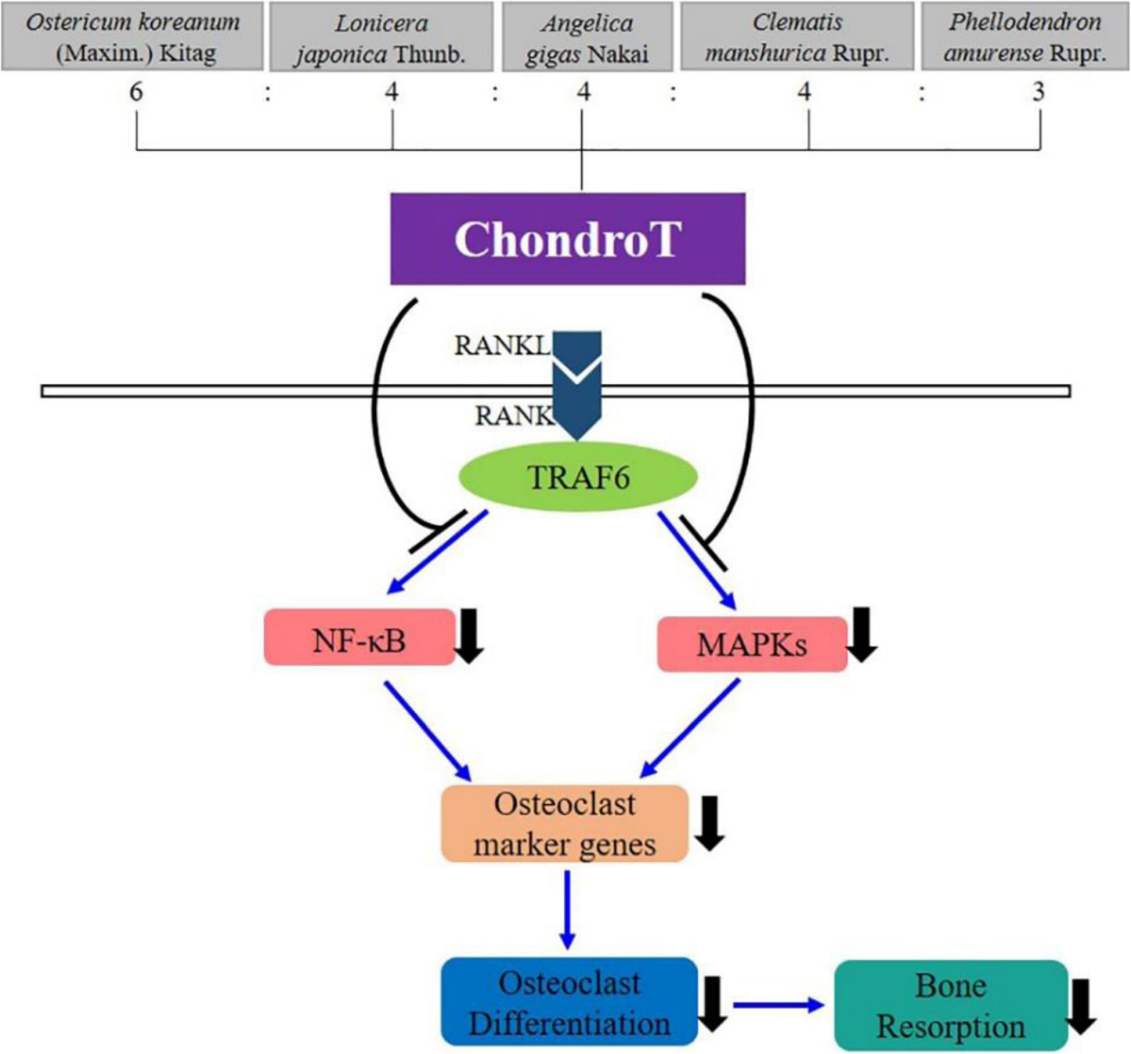
Schematic diagram for ChondroT regulation on osteoclast differentiation and function. RANKL binding to RANK on the surface of osteoclast precursors induces the recruitment of TRAF6, which leads to NF-κB activation and translocation to nucleus, as well as the phosphorylation of MAPKs. Both pathways could be inhibited by ChondroT or its constituent herbs, and subsequently inhibits the osteoclast-specific genes, thus resulting in the reduction of mature osteoclast with potent bone resorptive activity

In summary, the current study demonstrated that ChondroT could suppress RANKL-induced osteoclastogenic differentiation in BMDMs for the first time. As for the underlying molecular mechanisms, our results showed that ChondroT attenuated RANKL-stimulated activation of NF-κB and MAPKs pathways, subsequently resulted in the reduction of its downstream osteoclast-related proteins. These results contribute to the reduction of osteoclast formation and bone-resorptive function (Fig. 7). Our results indicate that ChondroT might be a novel therapeutic candidate for treating osteoclast-related bone diseases.

## Supplementary information

**Additional file 1.** Effects of *Phellodendron amurense* Rupr. on RANKL-induced osteoclast differentiation in BMDMs.

## Data Availability

Data and materials are available from authors on reasonable request.

## Abbreviations

A: *Angelica gigas* Nakai
AP-1: Activator protein 1
BMDMs: Bone marrow-derived macrophages
C: *Clematis manshurica* Rupr.
GHJTY: Ganghwaljetongyeum
L: *Lonicera japonica* Thunb.
MAPKs: Mitogen-activated protein kinases
M-CSF: Macrophage colony-stimulating factor
MMP9: Matrix metalloproteinase 9
MNCs: Multinucleated cells
MTT: 3-(4,5-dimethylthiazol-2-yl)-2,5-diphenyltetrazolium bromide
NFATc1: Nuclear factor of activated T cells c1
O: *Ostericum koreanum* (Maxim.) Kitag.
P: *Phellodendron amurense* Rupr.
RANKL: Receptor activator for NF-κB ligand
TRAF6: Tumor necrosis factor (TNF) receptor-associated factor 6
TRAP: Tartrate-resistant acid phosphatase
α-MEM: Alpha-minimum essential medium
