# Supplementary material for: Inhibitory effects of ChondroT and its constituent herbs on RANKL-induced osteoclastogenesis
Source: BMC Complement Altern Med. 2019 Nov 20;19:319. doi: 10.1186/s12906-019-2737-8 (PMC6864957; doi:10.1186/s12906-019-2737-8)
Supplement: Supplementary file 1 — Additional file 1. Effects of Phellodendron amurense Rupr. on RANKL-induced osteoclast differentiation in BMDMs. [file 12906_2019_2737_MOESM1_ESM.docx]

**Inhibitory effects of ChondroT and its constituent herbs on RANKL-induced osteoclastogenesis**

Rui Hong Guo^a^, Seon-Jong Kim^b^, Chan-hun Choi^b^, Chang-su Na^b^, Bok Yun Kang^a^, and Young Ran Kim^a^

^a^ College of Pharmacy, Chonnam National University, Gwangju 61186, Republic of Korea

^b^ College of Korean Medicine, Dongshin University, 185 Geonjae-ro, Naju-si, Jeollanam-do 58245, Republic of Korea

****Corresponding author***

**Young Ran Kim**, PhD, Professor

College of Pharmacy and Research Institute of Drug Development, Chonnam National University, Gwangju 500-757, Republic of Korea

Tel: +82-62-530-2923, Fax: +82-62-530-2949

E-mail: [kimyr@chonnam.ac.kr](mailto:kimyr@chonnam.ac.kr)

Fig. S1

a


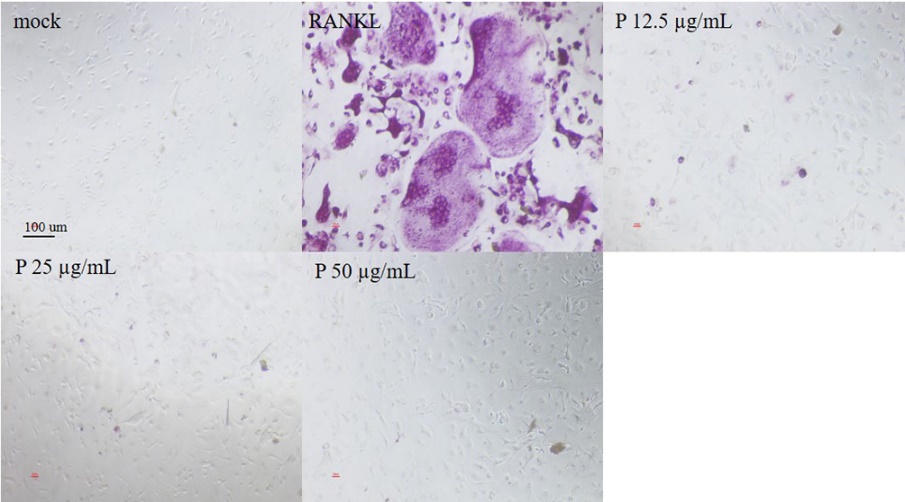


b


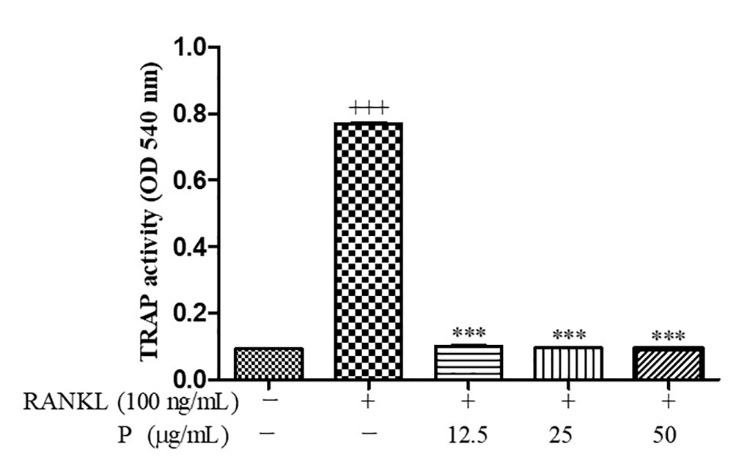


c


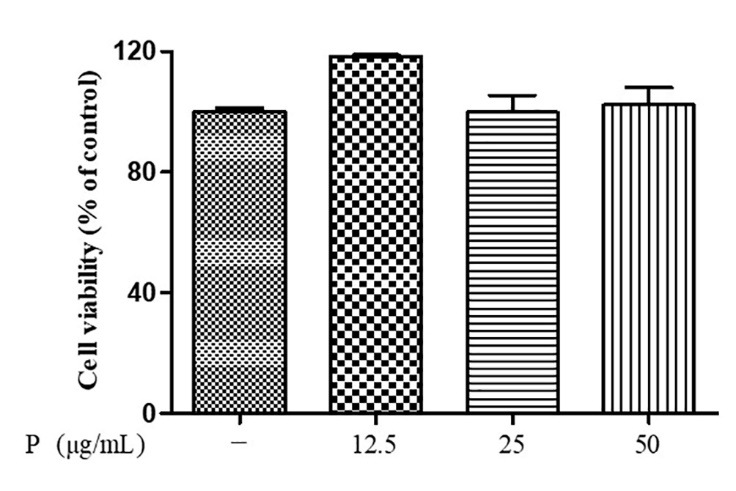


**Fig. S1** Effects of *Phellodendron amurense* Rupr. on RANKL-induced osteoclast differentiation in BMDMs. **a** TRAP-positive stained cells were stained with TRAP staining kit. **b** Dispense 30 µL of culture supernatant with 170 µL of chromogenic substrate/tartrate-containing buffer at 37 ℃ for 3 h. TRAP activity in osteoclast culture supernatant was determined at 540 nm. **c** BMDMs pretreated with *Phellodendron amurense* Rupr. (P) for 72 h were treated with MTT solution for 4 h, and then were dissolved in DMSO. Absorbance was read at 570 nm. ^***^*P* < 0.001 versus RANKL group; ^+++^*P* < 0.001 versus control group.
